# Traditional food plants of the upper Aswa River catchment of northern Uganda—a cultural crossroads

**DOI:** 10.1186/s13002-021-00441-4

**Published:** 2021-04-06

**Authors:** Eliot T. Masters

**Affiliations:** World Agroforestry (ICRAF), Nelson Marlborough Institute of Technology (NMIT), 322 Hardy Street, Nelson, 7010 New Zealand

**Keywords:** Agro-biodiversity, Underutilized species, Traditional food plants, Food security, Parkland agroforestry system

## Abstract

**Background:**

In the parkland agroforestry system of northern Uganda, smallholder farming households rely on a diversity of plant species to fulfil their nutritional requirements, many of which also serve a range of medicinal, cultural, and livelihood functions. The purpose of the study was to assemble an inventory of indigenous plant species used as food in four districts within the Aswa River catchment of northern Uganda, and to document their utilization and management by rural communities.

**Methods:**

From July 1999 to August 2000, a series of 61 community-based focus group discussions on the utilization of plant biodiversity were conducted in the vernacular language at 34 sites in four districts of northern Uganda, with participation by key informants self-selected on basis of their technical knowledge and personal interest. Of these, 232 respondents subsequently contributed to a collection of herbarium specimens, which were submitted to the Makerere University Herbarium for identification. On receipt of each specimen collected, a structured interview was conducted to document the botanical, ecological, seasonal, and alimentary attributes of each identified taxon, and details of its processing and utilization by the community from which it was obtained. The data analysis was undertaken during 2019 and 2020, including statistical tests to assess the relative importance of the cited taxa using the Relative Importance Index (RI), and to determine the similarity of edible plant use between the four cultures using the Jaccard Index of similarity (JI).

**Results:**

Key informant interviews yielded 1347 use reports (URs) for 360 identified specimens of 88 indigenous edible plant species. The data describes patterns of use of indigenous edible plants of four cultures of the Aswa River catchment of northern Uganda. RI scores ranged from 0.93 to 0.11, with fruit trees occupying the top 25 taxa (RI 0.45 and above). Jaccard similarity scores ranged from 25.8% between Lango and Acholi, to 15.8% between Acholi and Ethur, indicating that cultural factors appear to be more significant than shared ancestry as determinants of cultural similarity of plant use.

**Conclusions:**

The data constitute an inventory of on-farm plant species, including cultivated, semi-cultivated, and wild plants, integrated into a parkland agroforestry system in which useful trees and other plant species are sustained and managed under cultivation. Agricultural and on-farm plant biodiversity may be seen as a food security resource, and a nutritional buffer against increasing risks and stressors on low-input smallholder agriculture. Further studies should assess the intra-species biodiversity of these resources, with respect to farmer-valued traits and vernacular (folk) classification systems.

## Background

If the best time to plant a tree was 20 years ago, then there is no time like the past in which to document traditional technical knowledge.

This study is based on data collection undertaken during 1999 and 2000, as an inventory of species and a situation assessment of plant utilization and management in the parkland agroforestry system of northern Uganda, characterized by a range of landrace crops and a wide distribution of multipurpose tree species, which are selectively retained by farmers when fallow woodland is cleared for cultivation.

Globally, food plants have been assessed in context of agrobiodiversity (or agricultural biodiversity), a vastly inclusive category that includes the cultivated crop plants, semi-domesticated species, wild crop relatives and other, associated biota [1]—occupying trophic niches extending from soil mycorrhiza and other microorganisms to the ‘charismatic megaflora’ of parkland tree species of noted cultural import (such as the baobab, the shea butter tree, tamarind, and fig).

The indigenous plant biodiversity of Uganda has been well documented historically—classified and mapped according to vegetation type [2], and on the species level [3, 4]. Utilization of wild and semi-cultivated food plants has been considered in Uganda as a whole [5, 6], in specific regions [7], and in a number of regional studies [8, 9], some in northern Uganda [10], and one proximate to the study area [11].

According to Harlan’s reprise of the Vavilovian classification, northern Uganda lies within the ‘African noncenter’ of agricultural origin [12], distinguished by landrace cultures including finger millet (*Eleusine coracana* (L.) Gaertn.), *Sorghum bicolor* (L.) Moench, pigeon pea (*Cajanus cajan* (L.) Millsp.), cowpea (*Vigna unguiculata* (L.) Walp.), and simsim or sesame (*Sesamum indicum* L.), all of which comprise the major food crops of the study area, well recognized in the literature and addressed by agricultural policy and research.

The initial impetus for this study arose during a regional food shortage observed by the author in 1994, during which it was evident that households within the study area had little to fall back on during times of hardship [13], and that the elderly held—and passed on—a body of traditional technical knowledge of edible wild plants, which was observed to contribute to more favorable food security and nutritional outcomes in their communities. Significantly, this knowledge often involved the processing methods by which potentially harmful secondary compounds in the wild plants eaten only during times of hardship could be neutralized, rendering these ‘famine foods’ more palatable.

The aim of the broader study, conducted under the auspices of an integrated conservation and development project (ICDP) aimed at documenting and reinforcing sustainable use of the tree and other woodland plant species, was to compile a full inventory of plant species traditionally utilized by rural communities, drawing upon the expertise and interest of key informants, and leveraging tacit knowledge from those motivated to share it in order to codify utilization and management of plant resources. This was achieved through a series of multi-dimensional studies (cultural, ethnographic, socioeconomic, ecological, and botanical) of plant diversity, its use, and management by smallholder farming households across the project area, of which this paper presents the results relevant to edible plants.

The operational hypothesis driving the study was that use patterns of some plant species within and between the four cultures surveyed are similar, but that plant use within each culture bears a distinctive profile of plant use—and that, given that they inhabit similar geographies, the relative degrees of relatedness of edible plant use between the four communities might be expected to reflect ethnic as well as cultural dimensions.

## Methods

### The study area, communities, and landscapes

**Fig. 1:**
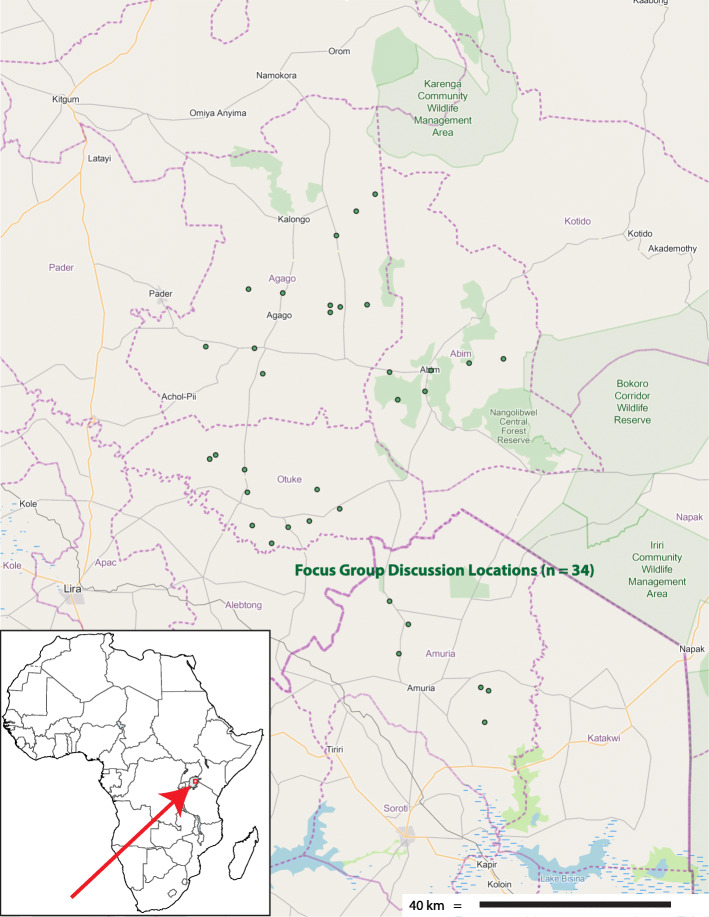
Map of the study area

The study area occupies the southern catchment of the Aswa River^1^, a major tributary of the Nile, which takes in the rivers Agago and Moroto as it descends the northern Uganda plateau to merge with its larger confluent at Nimule. Situated between 1 and 3° North, and from 33 and 34° East (see Fig. 1), the study area is characterized by wooded savanna standing on sandy loams overlaying a lateritic ironstone layer. Annual rainfall of between 800 and 1500 mm is unimodal, distributed between two peaks from April to September [14].

The peoples of the study area, residing in the four current districts of Otuke, Agago, Amuria, and Abim, represent four different cultures—the Acholi, Lango, Teso, and Ethur—each with a complicated and interlinked history of migration dating back over a thousand years—some details of which have been transmitted by oral tradition into living memory [15]. The inselberg ranges of the Agoro and Labwor hills, and the long massif of Mount Otuke (1600 m) provide geographic and cultural landmarks within oral tradition, from which a chronology of migrations has been derived according to the royal genealogies of Acholi. Although no such framework exists for the acephalous societies of the Ethur, Lango, and Teso, the historical famines recalled by all four cultures have been correlated to historical data recorded at the Rodah nilometer, near Cairo, which provides a chronology of eastern African rainfall over the past thirteen centuries [16], in confirmation of the oral histories.

There is a fundamental and audible distinction between the Ateso language and the other three languages of the study area—Acholi, Lango, and Thur—which share many words and structures, although the four tongues ultimately derive from the Nilotic branch of the Nilo-Saharan language group, estimated to be over 15,000 years old [17]. Whereas the oral histories of the Lwo^2^ cultures of Acholi and Ethur recall a migration from north to south, the Lango and Teso recall an east to west movement, the four groups ultimately settling together around Mount Otuke for a period, before dispersing into their current homelands [18]. Several hundred grinding pits can be observed on the granite face at the southern end of the mountain, along the Abim to Lira road; the author has been unable to locate any archeological assessment regarding the dates of these structures, which seem to indicate a substantial population residing in what today is a wilderness of deep cultural import.

While there is historical linguistic evidence for Lwo language being spoken in northern Uganda since before 1000 BCE [19], the migrations of the Lwo people are recalled to have begun in response to a drought dated to 1031–1058 CE. This event is remembered as resulting in a ‘breakup of the Nilotic cradleland’ along the Nile Valley [20] or the Bahr el Ghazal region of present day South Sudan, and sequential southern migrations of Sudanic and Nilotic peoples [21]. The Lwo people, common ancestors of the Acholi and the Ethur, seem to have moved south into the study area from the sixteenth century, while the ancestral Lango and the Teso migrated west from a homeland in present day Ethiopia, splitting off from the Jie clan of the Karimojong cluster between 1780 and 1840 [22]. The study area includes the eastern clans of Acholi and Lango (as indicated by the historical sub-regional designations East Acholi and East Lango respectively), comprised of clans distinct from their western counterparts in their enjoying more harmonious cross-cultural social relations, and, in the case of the Lango, by very different cultural and geographic origins [23].

While cattle genetic studies provide some triangulation regarding these human migrations [24], many of which can be linked to climatic events, for the most part cattle have been an intermittent and secondary factor in each of the four cultures of the study area. Each of the four cultures consider themselves to be agricultural rather than pastoral societies—‘people of the hoe’ for whom cultivation was supplemented by hunting, as reflected in many place names—Adwari signifying a place of hunting, other places indicating presence of prey, such as Bar Jobi, place of the buffalo. Historically, the cattle herds of the study area were devastated by several rinderpest epidemics from the 1890s, and again by a series of well-organized cattle raids a hundred years later. As a consequence, the first recorded observations of livelihoods in the study area, made during the early twentieth century, describe food systems based on cultivation, hunting and gathering of wild foods including plants, mushrooms, and termites [25, 26].

The communities of the study area are distinguished socially by widespread participation in community-based farming groups, many of them women’s groups, many of which are said to have been formed to facilitate distribution of relief food during a drought of the early 1980s. Culturally, these ‘self-help’ groups draw from traditional shared labor work groups, known in the Lango language as *wang tic* (for ‘burn the work’), in which groups of neighbors rotated between the household farms of their fellows, the group performing collective tasks to be compensated at day’s end with a shared pot of millet beer [25].

The landscapes of the study area are characterized by the parkland agroforestry system [27], in which useful tree species are conserved when land is cleared for cultivation, allowing for regeneration of woody species during fallow, and semi-domestication of some of these species through generational selection of farmer-favored attributes over time [28] (e.g., reliable yield, sweetness of fruit, palatability of leaves, and cooking or processing attributes of grains and pulses) [29]. The gathering, processing, and sale on local markets of woodland products, including wild foods, has been observed to be a gendered activity—the returns to which remain largely controlled by rural women in their custodial role within rural households [13].

### Data collection

Data collection began with a period of participative personal observation from 1992, with substantial time spent by the author in rural areas of the then Otuke County, Lira District, (now Otuke District), with the engagement, hospitality, and support of community-based groups. Formal research instruments were developed during 1999, and were administered from August of that year to September of 2000.

Sampling was undertaken as widely as possible within each of the four focus districts, each of which represents a different ethnicity and culture, during a time of increasing insecurity. The study area is typified by hamlets in which land use is traditionally allocated by the clan, and agricultural labor undertaken by groups of neighbors, of which many are formally constituted as farming groups, women’s groups, and youth groups.

The data collection was undertaken several years into an integrated conservation and development project which began in 1992, eventually engaging over 10,000 smallholder farmers affiliated to over 400 community-based groups in a range of program activities at the interface between plant biodiversity and rural livelihoods. This context allowed for mutual familiarity, credibility, and trust to be well established between the interviewers and respondents, and within the broader communities surveyed, and also provided opportunities for triangulation and confirmation of data during subsequent visits.

As a means of identifying key informants, the engagement of these community-based groups provided a logistical channel for the engagement of whole communities, and an invaluable social infrastructure, facilitating establishment of trust-based personal relationships between interviewers and respondents. On basis of their technical knowledge and personal interest in participation, key informants volunteered their time as subject matter experts on the foodways of tradition (*tekwaro*), and of the ‘early times’ (*ikare me con*), a pre-historical period known as the *aconya* (Lango, Acholi, Ethur) or *asonya* (Ateso).

From July 1999 to August 2000, a series of 61 focus group discussions on the topic were conducted in the local vernacular language at 34 sites (see Fig. 1), in order to introduce the study objectives, and to identify potential key informants. Discussions followed formal introductory meetings hosted by local leadership, with participation by one or more community-based groups of smallholder farmers representing 67 locations of origin (Local Councils, or LCs). Participants notably included the elders of an area, community leadership, and the members of one or more farming or women’s groups. Each exchange began with a general discussion of plant use, during which those with relevant knowledge and interest were encouraged to provide botanical specimens of plant species of interest.

Respondents were requested to provide botanical samples of food plants of interest to them, each of which was documented by completion of a questionnaire indicating the location, date, local names, seasonal availability, palatability classification (i.e., traditional food or famine food), habitat, plant type, part(s) eaten, a description or the plant, its harvesting and processing, storage time, and manner of consumption. Further information was recorded documenting the morphological characteristics, geographical distribution according to vegetation type for each specimen, according to its vernacular name.

Following their engagement on basis of prior informed consent, 232 key informants were identified on basis of prior specialist knowledge, their participation was determined by self-selection based on individual motivation and interest. With prior guidance on the requirements of botanical sampling provided during the focus group discussions, interested respondents were requested to collect one or more specimens of plants of interest for subsequent identification by the Makerere University Herbarium. Use of plant specimens as a focal point for structured interviews has been referred to as the ‘plant interview’ method of ethnobotanical research [30].

Each informant was interviewed individually, to complement the collection of one or more herbarium specimens. For each specimen collected, a separate sub-interview was conducted with the informant, and a separate entry was made on each specimen, for which multiple use reports were recorded by a single key informant. The data was recorded on interview sheets which were numbered sequentially. The results of each interview were subsequently digitized as a line in a spreadsheet shortly after the interview.

The study was conceived as an inventory of species use for the four districts, with a hypothesis that patterns of use would differ significantly between the four cultures represented therein, i.e., East Lango, East Acholi, Teso, and Ethur (Labwor). Data was recorded according to eight use categories—edible, medicinal, ritual, agricultural (including livelihood and environmental services), fodder (animal food), wood, and fuel (firewood and charcoal), and 52 sub-categories including 10 edible applications.

A distinction was attempted to be drawn between plant foods commonly consumed throughout the year or in season, and those foods which are only consumed in times of food shortage and hardship, due to their relative unpalatability and often rigorous processing methods required to reduce or mitigate the presence of anti-nutritional factors.

Botanical specimens were prepared by the researchers at the Shea Project offices in Corner Adwari and in Lira Town, and presented to the Makerere University Herbarium for identification by Latin binomial, for which local vernacular names were recorded during the interviews conducted in the four languages of the study area.

Taxonomic data were compiled on basis of contemporary botanical identification of each herbarium specimen as per the contemporary botanical nomenclature. Since that time, a number of species have been reclassified, necessitating verification of each identified species by cross-checking the original identification with the Plant List (http://www.theplantlist.org/), a comprehensive online database of plant names for all described plant species, maintained in collaboration between the Royal Botanic Gardens, Kew and the Missouri Botanical Garden [31].

### Data analysis

Given the differing sample sizes between the four districts, Spearman’s rho coefficient (*r*_s_) and statistical significance (*p* value) tests were run in order to determine whether there was a significant correlation between the number of respondents per district and the number of use reports per district, significant difference being defined as *p* < 0.05.

Relative Importance Index (RI) was calculated for each species, and separate RI values were obtained for the taxa cited by respondents from each district, in order to evaluate the relative importance of each species by culture, based on frequency of citation and number of use categories. Originally developed for ranking of medicinal plants by their pharmacological properties and body systems affected [32], the RI was subsequently adapted for the ranking of multiple-use species by use category [33].

$$ {\mathrm{RI}}_{\mathrm{s}}=\frac{{\mathrm{RFC}}_{\mathrm{s}\left(\max \right)}+\kern0.5em {\mathrm{RNU}}_{\mathrm{s}\left(\max \right)}}{2} $$

where RFC_s(max)_ is obtained by dividing the relative frequency of citation (RFC) for the species (FC_s_) by the maximum value in all edible species surveyed [RFC_s(max)_ = FC_s_/max (FC)], and RNU_s(max)_ is the relative number of use categories (RNU) for the species divided by the maximum number of use categories in all edible species surveyed [33].

In order to test the operational hypothesis, the Jaccard Index of similarity (JI) was calculated as an indicator of the relative commonality (or similarity) of edible plant citation between each of the four cultures [34]. This metric represents the relative degree of confluence (similarity) of plant utilization among and between the four cultures of the study area.

In considering the degree of similarity between any two populations, JI = *a* / (*a* + *b* + *c*), where *a* is the number of taxa cited by both populations, *b* is the number of taxa cited only by the first of the two populations, and *c* the number of taxa cited by the second of the two populations [35].

## Results and discussion

### Species diversity, relative importance, and cultural comparisons

Table 1 presents a summary of interview data and herbarium specimens collected from 232 respondents in 67 locations within the four districts, languages, and cultures of the study area. The key informant interviews yielded 391 edible use reports for 620 herbarium specimens, identified as 88 species of 66 genera within 36 plant families. Of the 36 plant families listed, the 10 most frequently cited families account for 58.3% of all use reports.

**Table 1:** Results Summary by District

District	Sub-Region	Locations of Origin	Culture	Number of Respondents	Respondents per location	Herbarium Specimens	%	Specimens per respondent	All Use Reports	Edible Use Reports
**Otuke**	East Lango	**21**	**Lango**	**84**	4	**247**	39%	2.94	552	**140**
**Agago**	East Acholi	**22**	**Acholi**	**62**	2.8	**148**	23%	2.39	343	**125**
**Amuria**	Teso	**17**	**Ateso**	**63**	3.7	**165**	26%	2.62	333	**76**
**Abim**	Labwor	**7**	**Ethur**	**23**	3.3	**70**	11%	3.04	119	**50**
	**Total:**	**67**		**232**	**3.5**	**630**			**1347**	**391**

Spearman’s rho coefficient was calculated as *r*_s_ = 0.8, the *p* value of 0.2 shows no correlation between the number of respondents by district and the number of edible use reports.

Table 2 presents the total number of use reports according to all use categories considered by respondents, indicating the relative importance of edible plants within each culture, comprising 29% of all use reports. While this paper considers only the edible uses, the total number of use reports is the basis for calculation of the RI of each species, as indicated in Table 3.

**Table 2:**
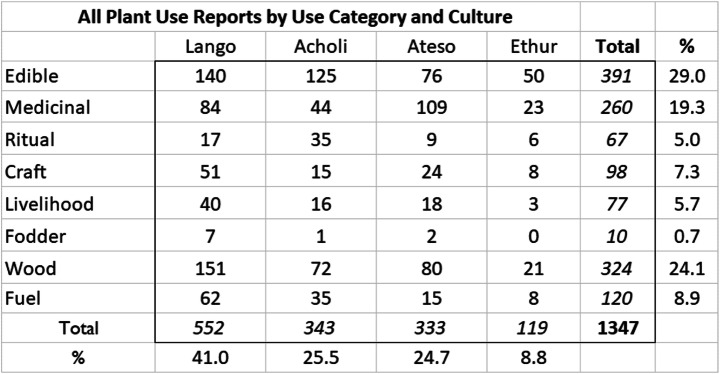
All plant use reports by use category and culture

**Table 3:** Edible Species of the Study Area

Family Genus	Species	Authority	Herbarium voucher number(s)	Plant form	Part consumed	RI	Seasonal availability	Vernacular ***Lango***	name ***Acholi***	***Ateso***	***Thur***
**Acanthaceae**											
*Asystacia*	*gangetica*	(L.) T.Anderson	SA39,103,130,197,291,467, 520	forb	leaf	0.34	mar-nov	Aconge, Aconge Wang-Gweno	Acwe	Ecoto	
*Justicia*	*exigua*	S. Moore	SA102	forb	leaf	0.11	mar-aug				Akweny-Akweny
**Amaranthaceae**											
*Achyranthes*	*aspera*	L.	SA92,117,324,500,561	forb	leaf	0.34	jan-dec	Kalamata	Ato Ite Oryang		
*Amaranthus*	*caudatus*	L.	SA364	forb	leaf	0.11	mar-dec	Ocobo			
*Amaranthus*	*graecizans*	L.	SA38,123,170	forb	leaf	0.19	mar-nov	Ocobo-Lango, Boyo	Boo	Eboo, Ekiliton	
*Celosia*	*trigyna*	L.	SA131	forb	leaf	0.11	apr-jun			Akelio, Ekiliton	
**Anacardiaceae**											
*Sclerocarya*	*birrea*	(A.Rich.) Hochst.	SA152,303,383;LA19,CE29, PO21	tree	seed	0.52	mar-aug	Ijakait	Titigo	Ejikai	Thibo
*Searsia*	*pyroides*	(Burch.) Moffett	SA53,124,134,218,264,496; DN414	tree	fruit	0.48	jun-aug	Awaya, Awaca	Awaca	Epwatet, Ewayo	Oyukwere
*Searsia*	*natalensis*	(Bernh. ex C.Krauss) F.A.Barkley	SA6,265,394;LA14,DN398	shrub	fruit	0.48	feb-mar		Rwa-rwa	Ewayo	Ozokwere
**Annonaceae**											
*Annona*	*senegalensis*	Pers.	SA21,120,137,191,223,259, 407,450;LA9,CE9,DN418	tree	fruit	0.89	apr-jul	Obwolo	Obwolo, Obolo	Ebwolo	Obwolo
**Apocynaceae**											
*Carissa*	*spinarum*	L.	SA12,96,138,187,210,247, 461;DN428	shrub	fruit	0.52	jan-mar	Acuga	Acuga	Emuriei, Emuriet	Achuga
*Pergularia*	*daemia*	(Forssk.) Chiov.	SA91,429	forb	leaf	0.15	mar-aug				Okuru
*Saba*	*comorensis*	(Bojer ex A.DC.) Pichon	SA25,125,188,254;LA30, DN480	vine	fruit	0.45	jun-sep	Komo	Pwomo	Emago	
**Arecaceae**											
*Borassus*	*aethiopum*	Mart.	SA442,547;PO35	tree	fruit	0.33	oct-dec	Tugo	Tugu	Eduku	Tugo
					shoot		apr-jun				
*Phoenix*	*reclinata*	Jacq.	SA221,DN441	tree	fruit	0.29	feb-apr	Otit, Tit	Otit	Esasa	
**Asparagaceae**											
*Chlorophytum*	sp.		SA321	shrub	tuber	0.11	oct-dec				
**Cleomaceae**											
*Cleome*	*gynandra*	L.	SA8,44,89,115,127,204,405	forb	leaf	0.41	mar-oct	Akeo	Akeo	Ecadoi	
*Cleome*	*monophylla*	L.	SA104,541	forb	leaf	0.15	mar-aug				Akeo-Jok
**Commelinaceae**											
*Aneilema*	*spekei*	C.B.Clarke	SA147	forb	leaf	0.11	mar-sep	Odipa Ikong		Ekoropot	
*Commelina*	*africana*	L.	SA90,464	forb	leaf	0.26	mar-aug	Ototo		Ekoropot	Othoto
**Compositae**											
*Aspilia*	*pluriseta*	Schweinf. ex Schweinf.	SA403	forb	seed	0.11	sep-dec				Obongno
*Bidens*	*pilosa*	L.	SA106	forb	leaf	0.11	mar-nov				Kamalara
					seed		oct-dec				
*Bidens*	sp.		SA435	forb	leaf	0.11	mar-jun				Okarama
*Crassocephalum*	*vitellinum*	(Benth.) S.Moore	SA40,SA293	forb	leaf	0.15	mar-dec	Apuruku			
*Schkuhria*	*pinnata*	(Lam.) Kuntze ex Thell.	SA169,481	forb	leaf	0.22	apr - dec			Kilorokwin	
**Cucurbitaceae**											
*Cucumis*	*aculeatus*	Cogn.	SA28,50,129,225	creeper	fruit, seed	0.23	sep-dec	Okwer	Okwer	Akobokobo	
*Cucumis*	*ficifolius*	A.Rich.	SA422	creeper	seed	0.11	nov-dec				Okwer-Gwok
*Curcurbita*	*pepo*	L.	SA29,190	creeper	leaf, fruit, seed	0.15	nov-dec	Okono			
**Dioscoreaceae**											
*Dioscorea*	*alata*	L.	SA43,184,226,255	creeper	tuber	0.23	jan-feb	Obato	Obato		
*Dioscorea*	*bulbifera*	L.	SA192,228,262	creeper	bulbil	0.19	nov-dec	Ogoko	Okoo		
**Ebenaceae**											
*Diospyros*	*mespiliformis*	Hochst. ex A.DC.	SA13,110,207,288,408,554, 555;CE16,PO36	tree	fruit	0.49	may-sep	Ikum	Cumu	Ekum	
**Euphorbiaceae**											
*Acalypha*	*crenata*	Hochst. ex A.Rich.	SA58,111	forb	leaf	0.15	mar-aug	Ayuu			
**Hypoxidaceae**											
*Hypoxis*	*angustifolia*	Lam.	SA57	forb	seed	0.11	mar-aug	Ocutu			
**Lamiaceae**											
*Hoslundia*	*opposita*	Vahl	SA7,141,206,211,276,549	shrub	fruit	0.37	aug-nov	Apurumur, Edudu	Tutu	Etapukon	
*Hyptis*	*spicigera*	Lam.	SA74,201,230,289	forb	seed	0.30	nov-dec	Amola	Lamola		
*Leonotis*	*nepetifolia*	(L.) R.Br.	SA182,273,400	forb	nectar	0.26	mar-dec		Nyamtigo	Ecika	Chukuchuku
*Vitex*	*doniana*	Sweet	SA1,49,185,263,459;CE1,DN401	tree	fruit	0.63	nov-dec	Owelo	Oywelo	Ekarukei	Welo
*Vitex*	*madiensis*	Oliv.	SA133,557;PO5	tree	fruit	0.40	nov-dec		Oyelo	Ekarukei	
**Leguminosae**											
*Bauhinia*	*thonningii*	Schum.	SA155,301,340,460;LA24, DN396	tree	fruit	0.73	dec	Ogali	Ogali	Epapai	
*Crotalaria*	*ochroleuca*	G.Don	SA62,94,231,286,433	forb	leaf	0.26	mar-dec	Ajalu	Lala		
*Tamarindus*	*indica*	L.	SA5,99,156,232,406,451;LA20,32;CE12,DN421	tree	leaf	0.81	mar-aug	Cwao	Cwaa	Epeduru	
					fruit		nov-dec				
*Vigna*	*subterranea*	(L.) Verdc.	SA33,54,78,250,456,482	forb	seed	0.30	oct-dec	Kali, Dokolo	Kali, Adyel-Adyel	Adokolet	
**Loganiaceae**											
*Strychnos*	*innocua*	Delile	SA20,139,159,236,S93,564; CE22,DN430	tree	fruit	0.59	apr-sep	Akwalwakwala	Alingkwalo, Lakwalo kwalo	Eturukuku, Eturugugut	Okuakwala
*Strychnos*	sp.		SA95,431,453	tree	fruit	0.33	oct-dec				Okwakwalo
**Malvaceae**											
*Corchorus*	*olitorius*	L.	SA148	forb	leaf	0.11	mar-sep	Otigo-Agywa		Atigo	
*Corchorus*	sp.		SA10, SA47	forb	leaf	0.15	mar - nov	Otigo agwya	Otigo-Apapal		
*Corchorus*	*trilocularis*	L.	SA189	forb	leaf	0.11	mar-dec	Otigo	Otigo-lum		
*Grewia*	*arborea*	(Forssk.) Lam.	SA359,PO34	tree	fruit	0.29	jan-mar	Amoni			
*Grewia*	*mollis*	Juss.	SA22,75,121,140,194,237, 390,454;LA1,CE21,DN437	tree	fruit	0.85	aug-oct	Opobo	Pogo	Eparis	Opobo
*Grewia*	*villosa*	Willd.	SA19,81,268,566	tree	fruit	0.30	aug-nov		Olukuru		Okura
*Hibiscus*	*cannabinus*	L.	SA128,195,285,426	forb	leaf	0.23	mar-nov	Amalakwang		Emalakany	
					seed		oct-dec				
*Hibiscus*	sp.		SA69,113	forb	leaf	0.15	mar-nov	Amalakwang-Toke			
					seed		oct-dec				
**Menispermaceae**											
*Cissampelos*	*mucronata*	A.Rich.	SA438	creeper	tuber	0.11	dec-jan		Aboce		
**Moraceae**											
*Ficus*	*dicranostyla*	Mildbr.	SA16,80,449,553,563;PO24	tree	leaf	0.45	mar-may	Ebu	Bito, Owii	Ebule	Owii
					fruit		jun-aug				
*Ficus*	*glumosa*	Delile	SA3,180,209,389,516;LA4, CE4,DN386	tree	seed	0.81	apr-jun	Okworo	Kworo	Ebiyong	Kworo
*Ficus*	*mucuso*	Welw. ex Ficalho	SA77,DN389	tree	fruit	0.29	nov-dec	Ibule			
*Ficus*	*natalensis*	Hochst.	SA261,LA7	tree	fruit	0.22	mar-jun		Kituba		
*Ficus*	*sur*	Forssk.	SA4,48,122,193,260,280; CE62,DN469	tree	fruit	0.59	apr-jun	Oduru	Oduru	Edulo, Edurokoi	Oduru
*Ficus*	*sycomorus*	L.	SA2,448,479;LA12,DN394	tree	fruit	0.55	apr-aug			Ecalawinyo, Emidit	Atielowinyo
*Ficus*	*thonningii*	Blume	SA112,158,392,517;LA8, CE25,60;DN420	tree	fruit	0.74	jun-aug	Ibule	Pwoo	Ebule	
*Ficus*	*vasta*	Forssk.	SA32,222,346;LA5,CE24	tree	fruit	0.69	apr-jun		Olam	Eborboriei	
**Myrtaceae**											
*Syzygium*	*guineense*	(Willd.) DC.	SA154,229,361;CE30,DN417	tree	fruit	0.41	mar-jun	Akalacer, Kano		Elecer	
**Olacaceae**											
*Ximenia*	*americana*	L.	SA17,60,86,119,235,272;CE6	tree	fruit	0.48	apr-aug	Olimo	Lalilimo, Olelemo	Ailama	Alimo
**Oxalidaceae**											
*Oxalis*	*corniculata*	L.	SA45	forb	leaf	0.18	mar-aug	Yat Leny			
**Pedaliaceae**											
*Ceratotheca*	*sesamoides*	Endl.	SA68,227,287	forb	seed	0.19	nov-dec	Alodi			
*Sesamum*	*calycinum* subsp. *angustifolium*	(Oliv.) Ihlenf. & Seidenst.	SA478	forb	seed	0.11	nov-dec	Otigo-Anino	Otigo Nino	Emalerait	
*Antidesma*	*venosum*	E.Mey. ex Tul.	SA52,283	shrub	fruit	0.19	aug-nov	Odugu-Kulo			
*Bridelia*	*micrantha*	(Hochst.) Baill.	DN438	tree	fruit	0.25	may-jun	Orweco			
*Bridelia*	*scleroneura*	Müll.Arg.	SA24,76,109,136,183,245,402, 458,484.548;DN395, CE19, LA17	tree	fruit	0.93	aug-nov	Orweco	Larweco	Erweco	
**Poaceae**											
*Dactyloctenium*	*aegyptium*	(L.) Willd.	SA344,434	grass	seed	0.15	oct-nov			Ewuduwudu	Achele
*Pennisetum*	*glaucum*	(L.) R.Br.	SA37	grass	seed	0.11	aug-dec		Raa		
**Polygalaceae**											
*Securidaca*	*longipedunculata*	Fresen.	SA31,330,353,417,495,552; CE48,PO31	tree	leaf	0.59	jan-dec	Ilila	Odyer	Ediol	Lilia
*Oxygonum*	*sinuatum*	(Hochst. & Steud ex Meisn.) Dammer	SA83,SA143,SA421	vine	leaf	0.19	mar-sep				Agwata-Remo
**Rhamnaceae**											
*Ziziphus*	*mucronata*	Willd.	SA35,398	tree	fruit	0.22	jan-dec				Langu
**Rubiaceae**											
*Fadogia*	*glaberrima*	Welw. ex Hiern	SA51,212,573	shrub	fruit	0.19	may-aug	Acetdyel			
*Gardenia*	*ternifolia*	Schumach. & Thonn.	SA271,477;LA2,CE50,PO14	tree	fruit	0.48	feb-mar		Odwong	Ekoroi	
*Sarcocephalus*	*latifolius*	(Sm.) E.A.Bruce	SA15,246,282,354,375,462; CE65,DN452	tree	fruit	0.74	nov-dec	Ibele	Oculup	Eutudolei	
*Vangueria*	*apiculata*	K.Schum.	SA84,101,224,395,585;PO27	shrub	fruit	0.37	mar-nov	Amalera	Adengonye	Emalere, Elepulepu	Omorokodo, Polere
**Salicaceae**											
*Flacourtia*	*indica*	(Burm.f.) Merr.	SA67,284	tree	fruit	0.22	mar-aug	Yat Dago, Kokowi			
*Oncoba*	*spinosa*	Forssk.	PO8,23	shrub	fruit	0.29	nov-jan	Ideloro			
**Sapotaceae**											
*Vitellaria*	*paradoxa* subsp. *nilotica*	(Kotschy) A.N.Henry, Chithra & N.C.Nair	SA23,42,200,258	tree	fruit	0.44	apr-jul	Yao	Yaa	Ekunguru	Yao
					oil		oct-mar				
**Solanaceae**											
*Physalis*	*minima*	L.	SA126,135,249,485,580	forb	fruit	0.34	aug-nov	Kongo Ogwal-Ogwal	Kongo Ogwal	Etagoli	
*Solanum*	*nigrum*	L.	SA11,118,198	forb	leaf	0.19	jun-nov	Ocuga	Ocuga, Ocugocuga		
					fruit		oct-dev				
**Vitaceae**											
*Ampelocissus*	*africana*	(Lour.) Merr.	SA14,186,233,457,562	creeper	fruit	0.26	sep-nov	Olok	Olok		
*Cyphostemma*	*adenocaule*	(Steud. ex A.Rich.) Desc. ex Wild & R.B.Drumm.	SA18,142,144,256,427,436, 514,558	creeper	leaf	0.45	mar-dec		Anunu	Emoros	Anunu
**Zingiberaceae**											
*Aframomum*	*alboviolaceum*	(Ridl.) K.Schum.	SA161,205,339,515	forb	fruit	0.23	nov-dec	Ocao		Acawoit	
*Aframomum*	*sanguineum*	(K.Schum.) K.Schum.	SA455	forb	fruit	0.11	nov-dec		Ocao		
**Zygophyllaceae**											
*Balanites*	*aegyptiaca*	(L.) Delile	SA36,82,162,384,545;CE13	tree	leaf, fruit, seed	0.45	apr-nov	Too	Too	Ecomai	Thoo

Table 3 lists the indigenous plant species used as food within the study area, providing the overall RI value of each species indicated, while Table 4 presents the ranked list species with an RI greater than 0.50. Species ranking by RI was further calculated for each of the four cultures of the study area. As a basis for cross-cultural comparison, Table 5 provides the twelve highest RI values for each of the four cultures. There results show a cultural confluence in the very highest ranked species, notably *Bridelia scleroneura*, *Annona senegalensis Grewia mollis*, *Tamarindus indica*, and several species of genus *Ficus*. Although cited by three of the four cultures, *Balanites aegyptiaca* was highly ranked only by the Ethur, possibly reflecting a cultural preference for the edible leaves.

**Table 4:** Edible Taxa with RI greater than 0.5

Rank	Genus	Species	RI
1	*Bridelia*	*scleroneura*	0.93
2	*Annona*	*senegalensis*	0.89
3	*Grewia*	*mollis*	0.85
4	*Tamarindus*	*indica*	0.81
5	*Ficus*	*glumosa*	0.81
6	*Ficus*	*thonningii*	0.74
7	*Sarcocephalus*	*latifolius*	0.74
8	*Bauhinia*	*thonningii*	0.73
9	*Ficus*	*vasta*	0.69
10	*Vitex*	*doniana*	0.63
11	*Ficus*	*sur*	0.59
12	*Securidaca*	*longipedunculata*	0.59
13	*Strychnos*	*innocua*	0.59
14	*Ficus*	*sycomorus*	0.55
15	*Carissa*	*edulis*	0.52
16	*Sclerocarya*	*birrea*	0.52

**Table 5:**
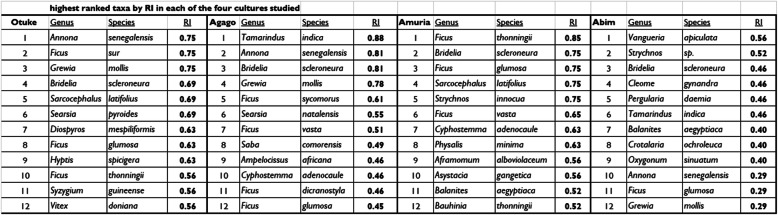
Twelve highest ranked taxa by RI in each of the four cultures studied

**Fig. 2:**
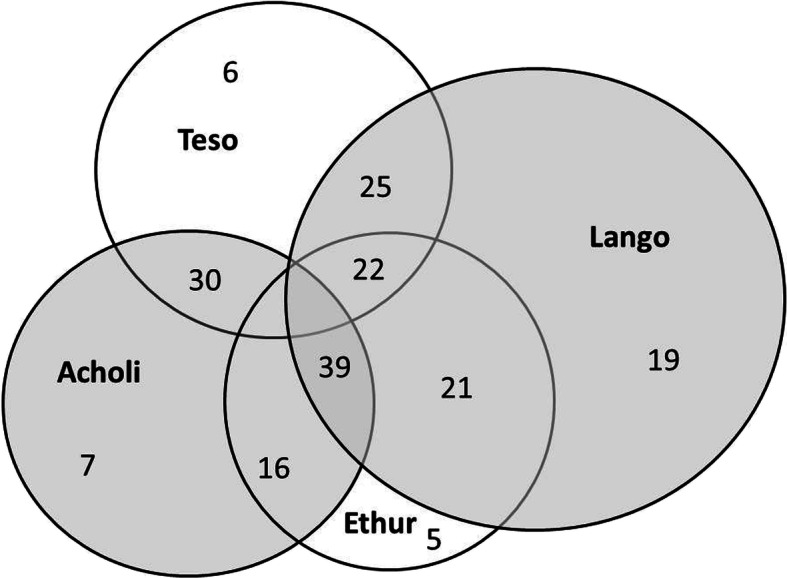
Edible taxa cited by language and inference *n* = 88

Figure 2 charts the edible taxa cited by each of the four cultures, indicating the number of taxa cited uniquely by each culture, and the number of cited taxa shared between each of the four cultures, as listed in Table 6. Using these figures, Table 7 provides the Jaccard Similarity Index for each cultural interface, ranging from 15.5% (Acholi and Ethur) to 25.8% (Lango and Acholi). Taken together, these values indicate relative commonality of edible plant use between cultures, providing a basis for comparison of one aspect of cultural similarity (see Fig. 2).

**Table 6:** Proportion of Edible Taxa by Culture and Interface

	Lango	Acholi	Teso	Ethur
Edible Taxa:	63	49	42	36
Lango	30%^a^	80%	60%	58%
Acholi		14%	71%	44%
Teso			14%	61%
Ethur				14%

^a^underlined figures indicate proportion of taxa cited solely by the given culture

**Table 7:**
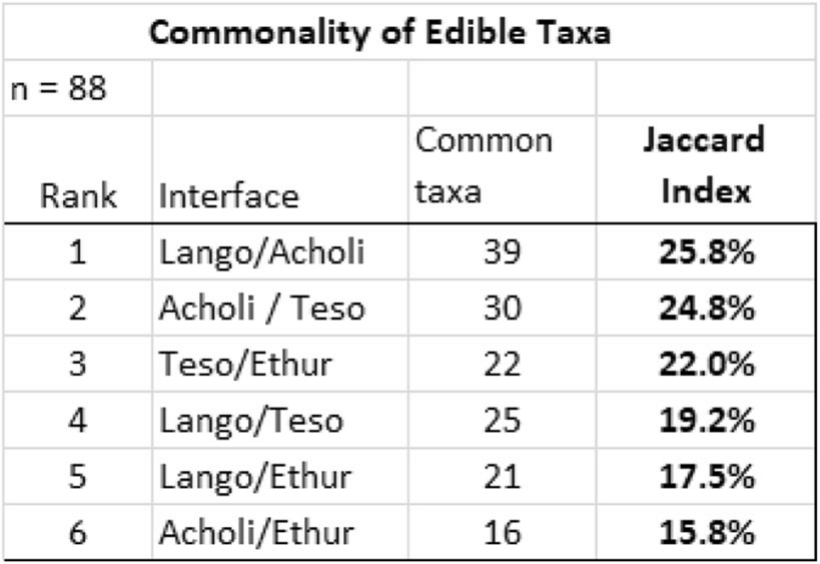
Commonality of edible taxa

Historically, the Acholi and Ethur (JI of 15.8%) share common (if temporally distant) ethnic origins, as do the Lango and Teso (JI of 19.2%), but the cultural divergence between each of these pairs is exemplified by their distinctly divergent languages. By contrast, the more recent cultural confluence between the Lango and Acholi (JI of 25.8%) is exemplified by the historical adoption by the Lango of a Lwo-based language quite distinct from their original tongue [22, 36]. These results could be interpreted as indicating that a more recent cultural affinity is a stronger determinant of cultural similarity than shared ethnic origins of the more distant historical past, but on the other hand, there is no direct cultural (nor geographic) proximity between the Acholi and Teso, despite their relatively high JI of 22.0%.

**Fig. 3:**
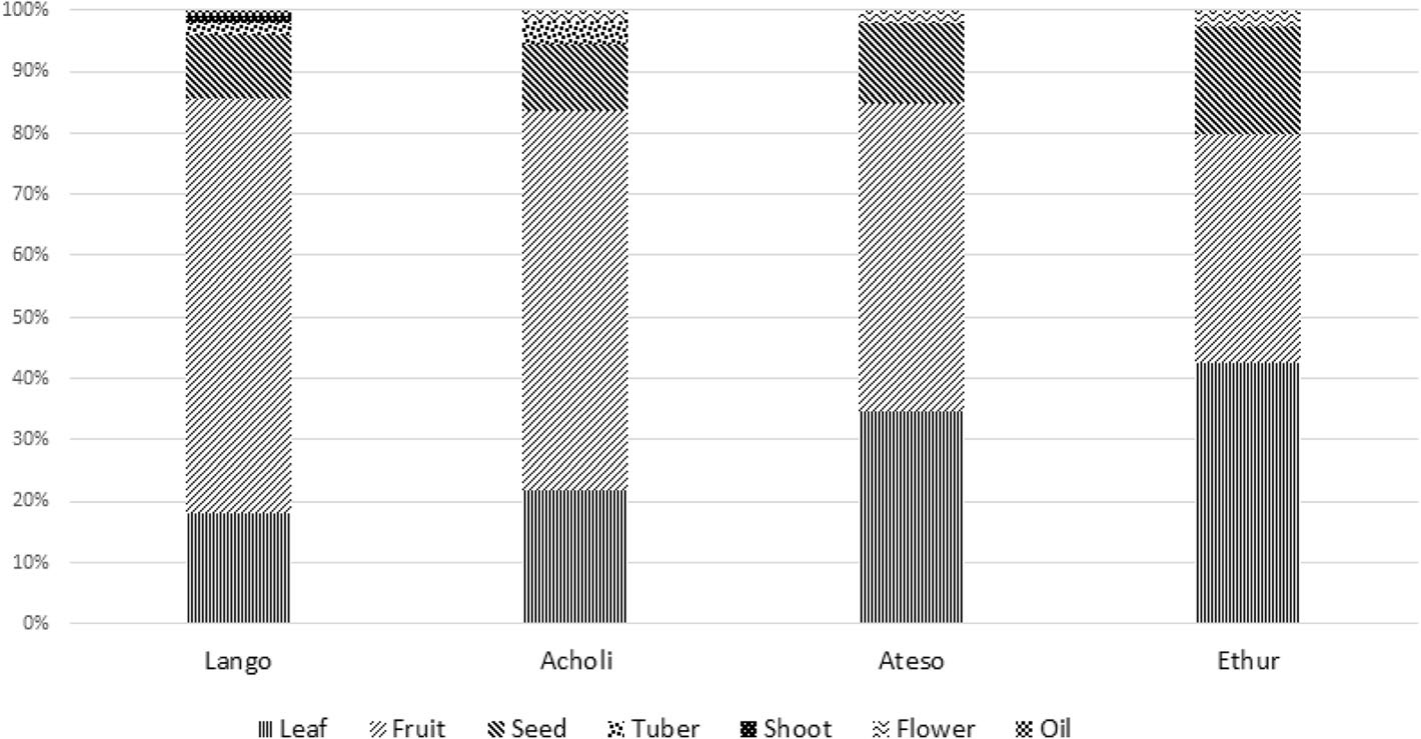
Proportion of edible use reports by plant part and culture *n* = 391

Drawing from descriptive statistics as a further basis for cross-cultural comparison, Fig. 3 shows the proportion of edible use reports by plant part for each of the four cultures. These results seem to indicate that leaf and seed protein are of relatively greater nutritional importance to the Ethur than the other three cultures, and that leaf protein is of higher importance to the Teso than to the Lango and Acholi, whose similar profiles are consistent with their notably high Jaccard similarity index.

### Species utilization

Drawn from 1447 use reports based on an herbarium collection of 630 specimens, the broader data set documents the nutritional, economic, medicinal, and cultural uses of 213 plant species, from annual herbs to trees, with local names recorded in the Lango, Acholi, Ateso, and Thur languages.

Noting that most species are used in multiple ways by respondents, the results describe the edible plants (29%, with 391 use reports); medicinal plants (19.3%, with 260); plants with cultural uses (18.7%, with 252) and plants utilized for their wood (24.1%, with 324) or as fuel (8.9% with 120 use reports). While only the edible plant applications are relevant here, the other use categories of application have been used to generate the RI value for each species, as described in the Methods section above.

The overall on-farm plant biodiversity of the study area includes cultivated and semi-cultivated species, many of which are indigenous, along a broad interface between cultivated plants, wild plants, and wilding crops from earlier rotations. The most heavily sampled species, *Bridelia scleroneura* with 13 specimens, yielded the greatest number of individual use reports—42, across each of the four broader categories, comprising two medicinal applications, six cultural uses, 21 use reports for the wood, and 13 edible use reports (all involving the fresh fruit).

Species used as food include plants bearing edible leaves, fruits (eaten fresh, or used in cooking), roots and tubers, and seeds, including commonplace cultivated species and those collected as wildings or weeds in cultivated fields, and gathered from woodland, wetland, and fallow. Not all of the taxa listed in Table 3 can be considered wild species *sensu stricto*. While graminoid seeds and leaves and fruits from trees and shrubs in particular are collected from the wild, indigenous plants which occur in the wild are sometimes cultivated as minor crops (often called underutilized or ‘orphan’ crop species), are considered here, including a range of leafy vegetables—*Amaranthus* spp., *Hibiscus* spp., African spider-plant (*Cleome gynandra*)—and seed crops including Bambara groundnut (*Vigna subterranea*), the wild sesame relatives *Sesamum calycinum* subsp. *angustifolium* and *Ceratotheca sesamoides*, and the ‘proto-sesame’ *Hyptis spicigera*.

To understand the convergent foodways of the four cultures of the study area, it is useful to consider the form of a typical meal. While porridge is often taken in the morning, an afternoon or evening meal is commonly based on a starch component, a pulse component, and a leafy green (vegetable component), based around finger millet, *Eleusine coracana*, a landrace crop, and a staple food. Finger millet grain is ground to a fine flour between stones, and stirred (‘mingled’) into boiling water to form a porridge, or, with further flour added, into a boiled bread (*kwon* in the Lwo languages, *atap* in Ateso), which outsiders might refer to as a ‘stiff porridge,’ though it is eaten as a solid, pasty ‘cup’ in which the relish is scooped by hand.

A standard meal combination would consist of the boiled bread eaten with ground pigeon pea over which shea butter has been drizzled. The same bread can be made with sorghum or maize flour, or can be substituted with mashed cassava tubers or sweet potato. This basic dish is served alongside a cooked green vegetable, or a vegetable sauce, depending upon palatability of the leaves.

### Plant parts used

#### Leaf vegetables

Respondent classification of leafy green vegetables is based on the distinction between the more unpalatable species, from which the secondary compounds must be removed, from others which may be ‘fried’ directly, without such prior processing. For greens with a more pronounced sour or bitter taste, the leaves are processed by parboiling in the ‘first water,’ which is discarded.

Notable on all types of ground throughout the study area are wilding or relict *Cleome gynandra*, which appears in cultivation from around March to October. Its leaves are characteristically bitter with secondary compounds, so the water in which it is parboiled is discarded prior to assembling the final configuration, in which the cooked leaves are combined with an *alodi* paste of sesame and groundnut to sweeten the sauce. The leaves may be served alone, or combined with *Hibiscus* spp., *Amaranthus graecizans*, *Curcurbita pepo*, or *Cyphostemma adenocaule*.

Likewise frequent as wilding or relict individuals are several types of *Hibiscus*, known generically as *malakwang* (Lango, Acholi, and Thur) or *emalakany* (Ateso), from which leaves are constitute a popular dish. Whereas *Cleome* is bitter, Hibiscus is characteristically sour due to its flavonoids and phenolics [37]; as with Cleome, the parboiling water is discarded prior to mixing with *alodi* paste, although fermented Hibiscus seeds known as *toke* were formerly used in the manner, as a means of sweetening the sauce.

While four of the samples collected were identified as *Hibiscus cannabinus*, commonly known as kenaf, two specimens were only identified as *Hibiscus* sp. Several other studies [5, 38] identify vegetable Hibiscus as *H*. *sabdariffa* (commonly known as roselle), from which the calyces are harvested for the tea known in Arabic as *karkade*. Katende et al. include *H*. *cannabinus*, *H*. *diversifolius*, as well as *H*. *sabdariffa* as malakwang species, with *H*. *acetosella* designated as *malakwang kulo* in Lango [6].

Vegetable species which may be eaten directly and without parboiling include *Crassocephalum vitellinum*, which is usually mixed with *Solanum nigrum*—in Lango (Otuke District); it is eaten alone only in times of famine. Other vegetables which may be fried directly include *Curcurbita pepo*; the young tender leaves are picked, the rough surface trichomes are removed, the leaf wilted, and then fried or boiled. Vegetables which require parboiling include *Cyphostemma adenocaule*, *Oxalis corniculata*, *Oxygonum sinuatum*, *Sesamum calycinum* subsp. *angustifolium* (the leaves of which are mixed with other vegetables to reduce the cooking time, and to ‘make better soup’), and *Solanum nigrum*.

*Commelina africana* is eaten alone, or mixed with *Hibiscus* spp. or *Corchorus trilocularis*, of which there are two types, commonly mixed; it is not eaten alone, but is combined with other leafy greens such as cowpea or *Crotalaria ochroleuca*, which is commonly interplanted with cereals and simsim (sesame), and may be boiled fresh, or dried and stored.

The young leaves of *Senna bicapsularis* are commonly eaten as a sauce in Abim, but are considered a famine food in Agago; in Amuria, *Senna obtusifolia* is described as ‘one of the traditional vegetables liked best by the Iteso,’ where the first cooking water is discarded ‘due to its black color,’ the leaves eaten with groundnut paste. In Otuke, it is boiled in water and ‘cooked down to dry,’ then added to a *Cleome* sauce, while in Agago, the leaves can be fried directly, as they are not considered bitter—a distinction suggestive of cultural or individual preference, with possible implication of intra-species diversity within the study area.

Trees which provide edible leaves include *Balanites aegyptiaca*, the young leaves of which are boiled twice (the initial water being discarded as ‘too sour’), pounded, ground, re-boiled, and mixed with groundnut paste. The species is considered a famine food by some respondents, but a palatable regular food by others (e.g., those of Abim). In Amuria, the tree is reportedly lopped to that young branches may develop, from which the tender new leaves are picked. According to respondents in Agago and Abim, the pounded young leaves of *Securidaca longipedunculata* are likewise prepared and eaten as sauce. Whether these distinctions reflect cultural rather than individual preferences is not clear.

Other trees and shrubs providing edible leaves include *Grewia mollis* (commonly combined with other vegetables, e.g., *Cleome* or *Hibiscus*) and *Justicia exigua*. In Amuria, the young leaves of *Tamarindus indica* are boiled, pounded, and boiled again, to make a ‘juice’ for flavoring porridge or bread (*atap*).

In Otuke, the young leaves of *Harrisonia abyssinica* are put into a sauce of pigeon peas, and given to a woman who has just given birth—but this is considered as a medicinal or ‘nutraceutical’ use, and not a regular food.

#### Fruits

A total of 48 fruit species were documented, providing a wide range of fruits mostly eaten fresh, particularly by children.

With a few notable exceptions, fresh fruits are collected from trees which have been conserved when cultivated land is cleared for fallow. Ripe fruits are usually gathered from the ground under the tree canopy, with the notable exception of *Syzygium guineense*—fruits picked from the tree by children, as the fallen fruits are normally rotten. Other fruit-bearing species include shrubs (e.g., *Carissa edulis*) and annual herbs (notably *Afromomum* spp. and *Solanum* spp.).

A number of fruits are commonly processed, some of which may be stored for extended periods, either pre- or post-processing. Of these, most are processed into pulp or juice and are eaten with porridge, in which a sour taste is appreciated—as when lemon juice is added to porridge made from finger millet flour.

Fruits eaten in this manner include *Aframomum alboviolaceum*, the fruit pods of which are crushed, its juice squeezed into flour for making porridge. Water is used to extract fruit pulp from the peeled fruit pods of *Tamarindus indica* (which can be stored for half a year or more); the pulp is then added to the flour from which a porridge or bread is mingled. Other fruits consumed this way include *Ficus* spp., *Searsia* spp., *Saba comorensis*, and *Strychnos innocua*.

Fruits which may be dried and conserved for later consumption include those of *Vitellaria paradoxa* subspecies *nilotica*, from which the fruit pulp may be formed into discs and dried as *yao adanya* (‘patted shea’ in leb Lango), a traditional delicacy which was commonly served, with shea butter, to honored guests. Rarely seen on local markets, the discs can be stored up to 2 years.

Of the several *Ficus* species cited, *F*. *sur* fruits are eaten fresh, or squeezed with fresh grass to make juice; they may also be dried and stored for later consumption. *Ficus glumosa*, *F*. *sycomorus*, and *F*. *thoningii* fruits may also be eaten fresh, or they may be dried. Other fruits which may be dried and conserved include those of *Cucumis aculeatus*, *Balanites aegyptiac*a, *Tamarindus indica*, and *Ziziphus mucronata*—the fruits of which may also be eaten fresh, or dried, and later pounded to separate the seeds from the seed-coat (pulp), which is used as a sweetener, sometimes added to porridge ‘as sugar.’

The fibrous coconut-shaped fruit of *Borassus aethiopum* may be beaten, to soften it, then eaten fresh, or grated and rinsed with water to make a drink; the harvested fruit can be stored for several months. In the mid-1990s, a concentrated *Borassus fruit* syrup was sold as a squash on the roadside at the Kafu river bridge; sweet, but not overly so, its taste was distinctively resinous, with a slightly soapy aftertaste suggesting the presence of saponins [39]. Similar products have been commercialized in Burkina Faso, and in Ghana [40].

Of the Cucurbitaceae, *Cucumis aculeatus* and *Curcurbita pepo* fruit may be dried and stored up to 3 months, kept separately from the dried leaves and seeds, which may be conserved for a longer time.

#### Seeds

While *Hibiscus* spp. have been considered above in the form of *malakwang* or *emalakany* (the vegetable sauce made from its tender leaves, harvested year-round ‘if there is rain’), in the dry season the vegetative plant is cut, bundled, and dried, the calyces threshed to obtain the seed, which can be stored up to 2 years prior to consumption.

Known as *toke* in all of the four language groups, the seed of *Hibiscus* was traditionally a stored food commodity of significance, used even as bride-price within the past few generations, following a series of cattle epidemics around the 1890s, which devastated the cattle herds [24]. Among the grandmothers of current adults were not a few ‘toke brides’ (A. Achen, personal communication 1992).

To prepare *toke*, the seeds are first dry-roasted, then allowed to cool before being rinsed with water, strained, and pounded into a puree or paste, which is boiled in water as a base for sauces; the watery remnant may be allowed to ferment before being used in place of the *alodi* paste to cook *malakwang* [41]. Fermented seed flour of *H. sabdariffa* features prominently in traditional diets of the poor in Sudan, where it has been described as a ‘meat substitute’ [42] analogous to the fermented proteinaceous flavoring product *dawadawa* in West Africa.

As noted above, the study area is within the ‘non-center’ of origin or diversity for sesame (*Sesamum indicum*) and its wild crop relatives [12]. Sesame in northern Uganda is widely considered a cash crop, and that as a food it is consumed not as an extracted oil, but primarily as a component of sauces and stews. This is also the case for the wild crop relatives of sesame *Sesamum calycinum* syn. *S*. *angustifolium* and *Ceratotheca sesamoides*, occurring as weeds or crop relicts in cultivated fields in the study area.

Another seed crop, which occurs wild and as a relict crop in cultivation and in fallow, is *Hyptis spicigera*, known in Lango as *amola* (*lamola*, Acholi), an ancient crop which has been classified as a ‘proto-sesame’ in western and central Africa [28]. Due to its similar form and alimentary function, *Hyptis* is closely associated with sesame, to which it is culturally associated [43] (if not botanically related), and with which may be conflated [44]. While consumption of the crop was not noted in Teso, it is well documented in Lango agricultural history [25]; Tarantino lists it among the first food crops planted by the Lango [26], and is known to be appreciated in the Madi culture of northwest Uganda [8] and in the Western Equatoria region of South Sudan [45]. *Hyptis* seed may be stored up to 2 years.

At maturity, stems are cut for dried in the compound, then and threshed and winnowed. Like simsim seeds pounded with ‘local salt and usual salt,’ the preparation of *Hyptis* in Lango and Acholi culture goes well beyond a generic paste. Following the dry roasting of the seeds, the half-cooked seeds are pounded to a flour using a mixture of conventional and vegetable salt, then mixed with warm water and molded into a saucer-shaped patties a hand’s breadth in diameter, called *agilgili* (Lango) or *akilikili* (Acholi), which is gently boiled into a semi-moist cake, following which sesame paste is added to the broth [5, 41].

This dish, which may be served with smoked beef (*olel*, Lango), is considered a traditional delicacy, and its place on the cultural menu is quite distinct; the taste of the *Hyptis* patty is pleasant, slightly nutty with a hint of umami, and despite the small size of the patty; it is very filling, and not more than two are likely to be eaten at a sitting (personal observations).

Actual sesame relatives which are eaten in a similar manner to that crop are *Ceratotheca sesamoides* and *Sesamum calycinum subsp. angustifolium*, both of which occur as wild plants or crop relicts, and may be inter-planted with cereals. While specimens of these plants were obtained only in Otuke and Amuria districts respectively, both were observed to be well-represented in the fields of Agago District during the 2019 agricultural season.

As with sesame, *Hibiscus* spp. and *Hyptis spicigera*, the mature stems are cut and bundled, let stand to dry, and then threshed and winnowed; seeds of both species are said to keep 1–2 years, and are pounded and then stone-ground into a paste which is served as is (as a type of *alodi*), or is used as a base for sauces.

Purely wild species providing edible seeds include the graminoids *Dactyloctenium aegyptium* and *Pennisetum glaucum*. Both grasses are cut and threshed to yield a grain which is ground into flour. In Amuria, the flour of *D*. *aegyptium* is mingled into boiling water as a porridge ‘when condition is serious’ (i.e., in times of famine), while in Abim the porridge may eaten with *Tamarindus indica* pulp.

In Agago, *P. glaucum* provides a boiled bread (*kwon*), and is sometimes fermented into beer. *D. aegyptium* is one of the more ancient grains harvested from wild species, known generically as *kreb* across the Sahara, with a notably high nutritional profile [46]—particularly in terms of the essential amino acids [47].

Other edible seeds include *Aspilia pluriseta*, *Bidens* sp. and *Cucumis figarei* (from which the seeds are roasted and ‘pounded with salt’ to form a paste), and *Hypoxis angustifolia*, the seeds of which are eaten fresh as a snack in Otuke.

Trees providing edible seeds include *Balanites aegyptiaca* and *Sclerocarya birrea*, from which the seed kernel is pounded and ground to paste as a base for sauces. In Amuria, the seeds of *Ficus glumosa* may be ground with cassava to make bread (*atap*) during times of hunger, while in times of hardship in Otuke, the dried seeds of *Ficus sur* are winnowed, then mixed with dried beer residue and ground into flour which is mingled into bread (kwon).

#### Tubers, bulbils, and shoots

Species from which edible tubers are obtained include two *Dioscorea* species, *D*. *alata* and *D*. *bulbifera*, of which the latter (also known as ‘aerial yam’) produces edible bulbils at the leaf axils. The list also includes one unidentified species, originally identified as belonging to the genus *Cissus*, but subsequently (if tentatively) identified as *Cissampelos mucronata*.

One species with edible tubers well documented elsewhere is *Hypoxis*, which was only noted for its edible seeds, although the bulb is reportedly considered edible by the Maasai and Kipsigis of Kenya [48]; at one cave site in South Africa, evidence for consumption of the cooked rhizome has recently been dated to 170,000 BP [49].

Beyond the above categories, other plant foods include the hypocotyl axis of the germinated seed of *Borassus aethiopum*, which is sometimes offered for sale on local markets or by the roadside. Germination may be stimulated in pits dug for that purpose, a practice also common in the Eastern Equatoria region of South Sudan [50].

##### Other plant foods

Plant foods other than the vegetative products mentioned above include the flower nectar of *Leonotis nepetifolia*, and the food oil shea butter, expressed from the seed kernel of *Vitellaria paradoxa* (as distinct from the solid fats of West African origins, the eastern subspecies *nilotica* yields an oil stable to about 20 °C).

### Seasonal availability

**Fig. 4:**
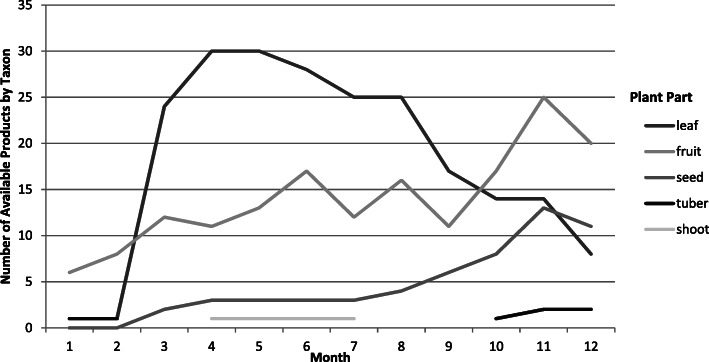
Seasonality of availability of product type (plant part)

The seasonal availability of each taxon is indicated in Table 3, and the availability of product types throughout the year (species per month by product type) is indicated in Fig. 4.

Seasonality data collected from each informant per species diverged in some cases, perhaps due in part to sub-regional ecologies including hydrological factors as well as rainfall patterns—noting again the precipitation gradient extending across the Aswa river valley, as described above. It is also possible that in some cases, informants indicated the occurrence of the developing plant within the growing cycle (particularly for annual forbs and graminoids), rather than the availability of harvested food products to which the question refers. For this reason, seasonality was triangulated against the literature [6].

Seasonality of edible plants can loosely be classified according to their occurrence in three separate seasons: the onset of rains (April through July), wet season (August through October), and dry season (November through March). By contrast to the complex seasonality of availability described by the data, the agricultural cycle is more narrowly pegged to the seasonality of precipitation. The availability of foods derived from agriculture corresponds to harvests undertaken during the wet season, roughly July to December, dried and stored foods being consumed through the dry season from January to June. The onset of rains at the end of the dry season, when food stores are depleted and agricultural labor (particularly women’s labor) is needed for planting and weeding the crops, is considered the ‘hungry season’—a period extending roughly from April to July.

Edible leaves tend to be harvested when young and soft, beginning shortly following onset of the rains. Edible leaves are harvested primarily from forbs (25 species) and creepers (2), which generally become available shortly after the onset of rains in March and April. Edible leaves are also collected from 4 tree species, from which young leaves from new growth are preferred, thus following a similar availability cycle to the annuals. While many leaves can be dried and stored up to 6 months, providing a potential buffer of availability during the dry season, freshly harvested leaves are significantly more palatable and nutritious than dried.

Edible fruit are gathered primarily from trees (21 species) and shrubs (7), and from forbs (4) and creepers (3). While storage of fruits is limited to a few exceptional cases (e.g., the processed and dried pulp of *Vitellaria paradoxa*, and the pods of *Tamarindus indica*), the availability of fresh fruits is evenly distributed throughout the year, with availability classified according to their availability as dry season or wet season fruits [41].

Dry season fruits, which mature alongside cultivated crops and are harvested concurrently, include *Aframomum* spp., *Balanites aegyptiaca*, *Bauhinia thonningii*, *Borassus aethiopum*, *Cucumis aculeatus*, *Gardenia ternifolia*, *Grewia arborea*, *Oncoba spinosa*, *Phoenix reclinata*, *Sarcocephalus latifolius*, *Solanum* spp., *Tamarindus indica*, and *Vangueria apiculata*.

More relevant to food security during the annual ‘hungry season’ are the wet season fruits, including *Ampelocissus africana*, *Annona senegalensis*, *Antidesma venosum*, *Bridelia* spp., *Carissa spinarum*, *Diospyros mespiliformis*, *Fadogia glaberrima*, multiple *Ficus* species (*F*. *dicranostyla*, *F*. *glumosa*, *F*. *sycomorus*, *F*. *sur)*, *Grewia mollis* and *G. villosa*, *Hoslundia opposita*, *Physalis minima*, *Saba comorensis*, *Searsia pyroides*, *Syzygium guineense*, *Vitellaria paradoxa*, and *Ximenia americana*.

Of these, the relative abundance and nutritional value of *Vitellaria paradoxa* was specifically mentioned by informants as a valued nutritional resource during the annual food deficit, when agricultural labor requirements are highest. The fruit is collected in the early morning by women and young children on their way to the fields, where the pulp will provide a snack during the work-day, the remainder and cleaned seeds carried home for de-husking and drying at the end of the day.

For species such as *Vitellaria paradoxa* with multiple edible uses, the availability of different products at different times of year provides another layer of nutritional diversity by edible use—fresh fruit from May to July, edible oil (and dried fruit) from October to March. Other notable examples include *Borassus aethiopum* (fresh fruit October to December, edible shoot April to June), *Ficus dicranostyla* (leaf March to May, fruit June to August), and *Hibiscus* spp. (fresh leaf from March to November, seed from October to December).

### Limitations of the study

Design of the study was somewhat ad hoc, partly as a result of overlapping areas of inquiry common to multiple studies serving the program objectives of a donor-supported integrated conservation and development project with an applied research component focused on on-farm biodiversity.

Although the state of ethnobotanical research at that time involved hypothesis testing and quantitative methods of data analysis which were largely based on informant consensus and ranking of species [51], the study was undertaken under the applied research component (on-farm biodiversity program) of an integrated conservation and development project (ICDP) with specific development-oriented objectives, as an inventory of species and their utilization, without regard to ranking.

As such, the purpose of the broader study was to document the ethnobotanical knowledge held by subject matter experts locally recognized for their expertise at the community level. The study did not aim to assess the awareness and knowledge of the broader population, nor generate any ranking of species by their relative importance.

As the core area of program emphasis was the Shea Butter Tree *Vitellaria paradoxa* subspecies *nilotica*, the study was conceived to supplement and complement the greater depth of data on that species. As an apparently compensatory consequence, relatively little attention was paid to that species by study informants, with only for specimens provided and only one medicinal application noted, against a vastly longer list of medicinal and cultural uses of the tree and its products documented through other project applied research [52].

The study was conceived as an inventory of species and their uses, and thus no attempt was made to assess preferences or rankings of the species, either within the respondent population or between cultures or locations. Respondents were not asked to list, nor prioritize, other food plants of significance beyond those for which they provided a herbarium specimen.

Botanical specimens were provided by the informant at the time of the interview, and as such the availability of this material determined in part the frequency of mention by individual informants. As a result, some plants were over-sampled (with more than a dozen vouchers for several species), while plants which were not easily sampled due to seasonality, or to physical limitations, may be under-represented in the collection. Although its value and use were very evident in the as multiple edible and other uses, just three specimens of *Borassus aethiopum* were collected—probably due to the impracticality of sampling the tree, in which the vegetative and floral parts are high above the ground.

During the data collection, it was not possible to draw a clear distinction between foods commonly consumed at present, in the past, and specifically in times of food shortage (‘famine foods’), since the personal tastes and circumstances of the informants could not be calibrated according to the data collection instruments.

Although it is tempting to read into the data, developing cultural generalizations (‘people of Labwor eat *Balanites* leaf and *Dactylotenium* porridge often, while those of Lango consider them famine foods’), there is insufficient data here to justify such conclusions.

Beyond the inventory of species by edible use, there were inconsistencies in the classification of species by designation, i.e., as a former food of the ‘old times’ (*ikare me aconya*), a famine food, or ‘common today’); there was likewise a degree of subjective disagreement regarding a species within the same district as regards habitat, abundance, establishment and even plant type (herb, creeper or vine—tree or shrub?), which prevents any meaningful consideration of these attributes.

Over the year of the study, each of the four interviewers displayed a different ‘style’ of data collection, with varying degrees of detail provided—some respondents seem to have been more forthcoming at sharing details of tradition and culture than others, possibly depending upon the tenacity of the interviewer, as well as factors such as gender and ethnicity, and the personality and mood of both interviewer and respondent.

Phenotypic diversity between sampled individuals was not evaluated, nor were questions asked about local classification of product types or favored characteristics of a given product. Areas of potential for further study include the intra-species biodiversity of the inventoried species, and how it is classified according to farmer selection criteria.

Contemporary studies of diversity and use of *Vitellaria paradoxa* subspecies *nilotica* within the study area revealed traditional classification systems by which the morphological and organoleptic attributes of shea fruit were known in the four languages of the study area [53]; a subsequent study provided an expanded this ‘folk classification’ framework to include the West Nile region [54]. Diversity in the morphology, proximate composition, and nutritional parameters of *Tamarindus indica* fruit and seed from different geographic origins of Uganda, including the study area, has also been assessed as significant [55–57].

Clearly, there is scope for assessment of the morphological, phenotypic, and nutritional parameters of intra-species diversity of the food plants presented here, preferably linked to locally and culturally specific evaluative frameworks.

## Conclusions

The data describe patterns of use of plants used as food in four districts of northern Uganda, including an inventory of species consumed by plant part, type of food, and seasonality. This study documents an inventory, and perhaps an archive, of traditional food plants—the use of which may or may not have changed during the last two decades since it was compiled.

Subsequent to the study, civil conflict brought wholesale displacement of communities in much of northern Uganda, including Agago and Otuke districts in particular, suffering massive social and cultural disruption as entire communities were displaced to military camps for half a decade [58, 59]. As many elders did not survive the hardships of war, and the youth who emerged from the camps 5 years later had no eagerness to return to subsistence agriculture, these events disrupted the chain of generational transmission of technical knowledge in a precipitate process of cultural erosion. The data presented here may thus have been drawn from a more extant body of traditional knowledge than that of today.

The extent to which the identified species are currently used as food is unknown. But there are recent indications that the plant species described here are still strongly represented on the farms of the study area. Observations made by the author within the study area during 2019 indicate both negative and positive impacts of the war and displacement on the ecological integrity of the agroforestry parkland. Many large, mature trees were cut to meet urban charcoal demand during the war, but a cohort of young trees established itself during the years of displacement which has been protected into maturity by returning communities. Under the canopy, and throughout cultivated fields, traditional food plants remain well represented in the cultivation mosaic, though relatively scarce on local markets.

The results of this study are intended as a reference for policy-makers engaged in support to agricultural systems in northern Uganda, and as a resource for further development of the plant foods described herein as a nutritional buffer serving systemic resilience at the local, national and regional levels.

It is hoped that diffusion of these results back to the informant communities may serve as a codified record of this knowledge, which might provide a measure of encouragement to farmers interested in the ancestral crops and wild foods described herein.

## Data Availability

The data from which this study is drawn will be freely shared with interested researchers by request.

## Abbreviations

BCE: Before the Common Era
BP: Before the Present
FAO: Food and Agriculture Organization of the United Nations
JI: Jaccard Index of similarity
RI: Relative Importance Index
UR: Use report

## Footnotes

1 Also written as Acwa, Acwaa, Achwa or Acaa

2 Also written as Luo
